# Clinical care review systems in healthcare: a systematic review

**DOI:** 10.1186/s12245-018-0166-y

**Published:** 2018-02-08

**Authors:** Laura E. Walker, David M. Nestler, Torrey A. Laack, Casey M. Clements, Patricia J. Erwin, Lori Scanlan-Hanson, M. Fernanda Bellolio

**Affiliations:** 1 Department of Emergency Medicine and Health Sciences Research, Mayo Clinic, 200 First St. SW, Rochester, MN 55905 USA; 2 Mayo Clinic Libraries and Health Sciences Research, Mayo Clinic, Rochester, MN USA

## Abstract

**Background:**

Clinical care review is the process of retrospectively examining potential errors or gaps in medical care, aiming for future practice improvement. The objective of our systematic review is to identify the current state of care review reported in peer-reviewed publications and to identify domains that contribute to successful systems of care review.

**Methods:**

A librarian designed and conducted a comprehensive literature search of eight electronic databases. We evaluated publications from January 1, 2000, through May 31, 2016, and identified common domains for care review. Sixteen domains were identified for further abstraction.

**Results:**

We found that there were few publications that described a comprehensive care review system and more focus on individual pathways within the overall systems. There is inconsistent inclusion of the identified domains of care review.

**Conclusion:**

While guidelines for some aspects of care review exist and have gained traction, there is no comprehensive standardized process for care review with widespread implementation.

**Electronic supplementary material:**

The online version of this article (10.1186/s12245-018-0166-y) contains supplementary material, which is available to authorized users.

## Background

Clinical care review is the process of retrospectively examining potential errors or gaps in medical care, with a goal of future practice improvement. This goes by many different names, sometimes with different audiences or case types, including peer review, adverse event review, sentinel event review, and root cause analysis. The concept of care review is widely accepted and encouraged among safety and quality healthcare leaders. However, a paucity of literature exists discussing and describing the current state of clinical care review.

The challenges and risks of contemporary medical care are well described. Medical error and its resulting outcomes have been defined and measured in many different ways, leading to varying quantifications of the effects [1]. The Institute of Medicine’s (IOM) 1999 report entitled “To Err is Human” [2] estimated that as many as 44,000 to 98,000 deaths annually in the USA occur as a result of medical error. Publication of “To Err is Human” was a landmark event in the recognition of the role of adverse events in medical care in the USA. This represents a shift in the focus on adverse events to look toward systems issues as a cause or error and a call to identify and act to prevent medical error. The National Quality Foundation estimates that, in 2010, medical errors affected 15.5% of Medicare beneficiaries, with nearly half of these errors considered preventable [3]. More recently, Makary and Daniel estimated that as many as 250,000 deaths per year in the USA are due to medical error, making it the third leading cause of death by their estimation [1]. Review of adverse events allows for investigation into, and classification of the causes of, the event and presents an opportunity to modify systems and behaviors to prevent future similar errors. As a part of the strategic approach for increasing safety, the IOM’s “To Err is Human” recommended “Identifying and learning from errors by ... encouraging health care organizations and practitioners to develop and participate in voluntary reporting systems.” They went on to say “Such systems can focus on a much broader set of errors, mainly those that do no or minimal harm, and help detect system weaknesses that can be fixed before the occurrence of serious harm, thereby providing rich information to health care organizations in support of their quality improvement efforts” [1].

Given the long standing call for clinical care review, with limited literature to inform care review systems, we conducted a qualitative systematic review to identify characteristics discussed in existing models for care review. The objectives are to (1) describe the current state of care review and (2) identify elements from published care review systems that contribute to their success. This systematic review will allow for a more complete evaluation of the current state of clinical care review and will identify areas for future scholarly activity.

## Methods

This is a qualitative systematic review of studies describing and evaluating care review systems. This study was exempt from our IRB review. This report adheres to the recommendations made in the preferred reporting items for systematic reviews (PRISMA) statement [4]. A protocol was written before the beginning of the investigation.

We included original research studies with any methodological design including cohort studies, case controls, and randomized trials, as well as commentaries, narrative reviews, letters to the editor, and abstracts in peer-reviewed journals that reported models for care review. Search results were limited to publications after January 1, 2000, to focus on publications since the release of “To Err is Human” [1]—a turning point in the way adverse events are analyzed and regarded. In choosing relevant publications, some articles described their process as the main purpose of the article, while others incidentally described a care review process, while instead focusing on a specific intervention or aspect of their mechanism for review. Either was acceptable, as they both shed light on a review system for analysis.

All types of patients and hospital settings were included, as well as recommendations from professional organizations and companies. This study’s investigators are physicians with involvement in quality improvement, adverse event identification and management, patient safety, and leadership of committees for clinical care review.

A senior expert librarian (P.E.) designed and conducted a comprehensive search of eight electronic databases, including Ovid MEDLINE, Ovid EMBASE, EBSCO CINAHL, Ovid CENTRAL, Ovid Cochrane Database of Systematic Reviews, Web of Science, and Scopus. Our search was done on June 10, 2016, and includes publications from January 1, 2000, through May 31, 2016. We included published conference abstracts in our search. There was no language restriction to the search strategy. Bibliography and reference lists of the articles obtained through database search were reviewed to identify additional publications for inclusion. The search strategy can be found in the Additional file 1.

### Qualitative assessment and data abstraction process

Two investigators (L.W. and D.N.) identified common domains in the initial literature review to determine which data to abstract, and included additional variables determined to be clinically important based on their experience in the clinical care review process and practice improvement. Domains included were description of systems improvement, educational output and feedback, description of a standardized process and referral mechanism, consideration of the case outcome, deliverables of the review system including non-punitive process and recognition of excellence, multidisciplinary involvement, dedicated process leadership, reviewer training, case blinding/anonymity, and implementation of improvement recommendations by the investigating group. These are further described in Table 1.

**Table 1:** Descriptions of the 16 domains of care review

Domain	Description
Systems analysis	Focused discussion on the role of the care system in the event being analyzed.
Functional department	The environment in which the care review process is deployed is in a position to receive the output from the review process and work toward implementation of change. There must also be buy-in from the stakeholders within the department (leadership, providers, nursing, ancillary staff).
Educational output	Process to create and disseminate lessons learned.
Standardized process	A clear flow of case identification and review that is consistently utilized.
Structured case classification	Consistent review process and inclusion of the same evaluations for all cases being discussed (e.g., use of a scaled rating system).
Feedback from and to the team	Individuals caring for patients who have had an adverse event are queried for their impressions and recollections of the incident, affording a glimpse of the decision-making process, and the state of the system at the time of the event. Following the review, they receive appropriate feedback regarding performance and systems issues to better understand all aspects to the event.
Human factor assessment	Contextual factors that affected decisions made by the care team are discussed in an effort to better understand the effects of the system on the individuals involved in the event.
Outcome consideration	The effect of the event on the patient is considered.
Non‐punitive	The review is explicitly and consistently identified as non-punitive and is not intended as a venue to mete out punishment to individuals for adverse events.
Recognition of excellence	Positive events during the event of care are identified and acknowledged.
Referral process	A voluntary referral process and/or an automated trigger (e.g., transfer to higher level of care) is available to identify cases for review.
Multidisciplinary	Inclusion of staff in a variety of roles (provider, nurse, etc.) in the review process to obtain multiple perspectives on the episode of care.
Process leadership	There are identified individuals to manage the process of care review consistently.
Reviewer training	Introductory explanation to reviewers of the ground rules of the process and how to think about the cases from appropriate perspectives (systems, care provided, team, human factors, etc.).
Case blinding	Cases are presented anonymously to minimize bias by reviewers.
Implementation of improvement recommendations	Care review group or committee is responsible for development and implementation of process improvement.

In phase I of the review, one investigator (L.W.) independently screened all titles yielded by the initial search strategy for possible inclusion. After identifying appropriateness for possible inclusion, phase II consisted of two reviewers (L.W. and D.N.) independently evaluating the abstracts of publications identified in phase I. The publications from phase II were then retrieved in full text and assessed for inclusion of domain abstraction in phase III by two independent reviewers. The agreed-upon articles were assessed by independent reviewers in duplicate, to abstract the identified domains of care review in phase III.

In phase II, disagreement between reviewers was reconciled by discussion and consensus. The investigators were not blinded to the authors, journals, or results of studies. In phase III, disagreements on the data abstraction were resolved by a third independent reviewer who assessed the article and determined if the theme was included in the care review process. Descriptions of the 16 domains were supplied to all reviewers prior to data abstraction for reference.

Critical appraisal is the process of systematically examining research evidence to assess its validity, results, and relevance before using it to inform a decision. Instruments developed to support quality appraisal usually share some basic criteria for the assessment of qualitative research. These include the need for research to have been conducted ethically, the consideration of relevance to inform practice or policy, the use of appropriate and rigorous methods, and the clarity, coherence of reporting, address of reliability, validity, and objectivity [5].

In considering the most appropriate instruments to use for critical appraisal, we considered using the Cochrane Collaboration Bias Appraisal Tool [6] and a modified Newcastle-Ottawa Scale tool [7]. The nature of our qualitative data abstraction precluded the use of these tools. While the studies we evaluated may have included randomized controlled trials and been at risk for bias, the results of the publications evaluated were not typically relevant to our goal of qualitative domain abstraction. Many publications we evaluated were narrative in nature—describing a process without presentation of data, either qualitative or quantitative. For those publications that did present data relevant to our domains, the effect of bias within the study was felt to be unlikely to impact our qualitative data collection because the abstracted domains—descriptions of processes—were not affected by the results of the studies. We reviewed the items described in the Standards for Reporting Qualitative Research (SRQR) [8] and the Enhancing Transparency in Reporting the Synthesis of Qualitative Research (ENTREQ) [9] statement. The SRQR and ENTREQ aim to improve the transparency of all aspects of qualitative research by providing clear standards for reporting qualitative research. When assessing the risk of bias, we decided not to exclude articles based on their quality assessment. All potentially valuable insights were included. From each study, we extracted the domains relevant to care review processes. We tabulated the results and created graphics based on frequencies. No quantitative data was appropriate for abstraction, so we did not perform a meta-analysis.

## Results

The initial library search strategy identified 1318 titles for review. In phase I, 440 abstracts were reviewed, 76 of which were selected for full-text review in phase II. Fifteen articles from outside sources and bibliography review were also identified and reviewed. In total, 91 full-text articles were assessed, and after reconciliation between two independent reviewers, 47 articles were initially found to be appropriate for inclusion in our analysis of the domains of care review. One article was removed in the abstraction process, as both reviewers independently determined that it did not meet inclusion criteria [10] leading to 46 unique articles reviewed.

**Fig. 1:**
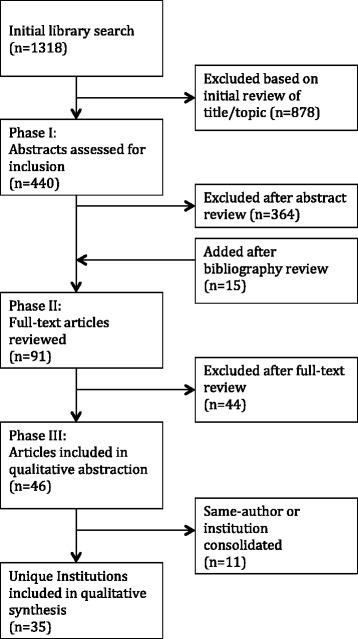
Study selection process

Domains were abstracted by two independent reviewers for each of the 46 articles in phase III. Articles that described a care review process from the same institution were consolidated to reflect the most complete view of that process possible, as aspects may have been reported differently in multiple articles/abstracts. Figure 1 shows the study selection process. Ultimately, we evaluated the care review systems from 35 unique institutions.

### Study characteristics

Among the 46 studies, 35 represented unique institutions and 11 were same authors/institutions describing different aspects of the process or domains. The types of articles identified included 14 descriptive [11–24], three editorials [25–27] 15 prospective [28–42], seven quality improvement projects [12, 43–48], and ten retrospective [11, 30, 49–56]. The 16 domains of successful care review that were identified for abstraction are presented and defined in Table 1.

**Fig. 2:**
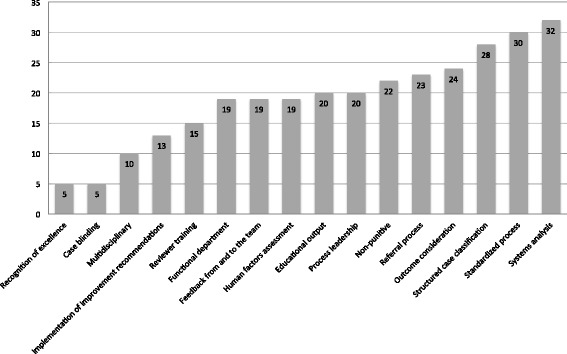
Frequency of domain identification

The percentage of frequency of each component is shown in Fig. 2. The most commonly identified component of a care review process was utilizing an analysis of systems issues contributing to the case (32 institutions, 91.4%), followed by utilizing a standardized process for case review (30 institutions, 85.7%) and use of a structured case classification system (28 institutions, 80.0%). The least common components identified were recognition of excellence and use of case blinding/anonymity in reviews (5 institutions, 14.3%). Some articles were consistent with more than one article type and were classified as both.

Table 2 shows the distribution of all components in the full-text articles reviewed, with consolidation of same-institutions. No article/institution identified all 16 items evaluated by reviewers. Two institutions identified 14 of 16 items: Lehigh Valley and Johns Hopkins.

**Table 2:** Distribution of care review domains

Author	Systems analysis	Functional department	Educational output	Standardized process	Structured case classification	Feedback from and to the team	Human factor assessment	Outcome consideration	Non-punitive	Recognition of excellence	Referral process	Multidisciplinary	Process leadership	Reviewer training	Case blinding/anonymity	Implementation of improvement recommendations	Number of components identified
Agee	X	X	X	X	X	X	0	X	0	0	X	0	X	X	0	X	11
Bender	X	0	X	X	X	0	0	X	0	0	0	0	0	0	0	0	5
Berk	0	X	0	0	X	X	0	X	0	0	X	0	0	0	X	0	7
Branowicki	0	X	X	X	X	0	0	X	X	X	0	0	X	X	0	X	10
Chan	X	X	X	X	X	X	0	X	X	0	X	0	X	X	X	X	12
Corcoran	X	0	0	0	0	0	0	X	X	X	0	X	X	X	0	X	8
Diaz	X	X	0	0	X	X	X	0	0	0	X	0	X	X	0	X	9
Edwards	X	X	X	X	X	0	X	X	X	X	X	X	X	X	0	0	13
George	0	0	0	0	0	X	X	0	X	0	0	0	X	X	0	0	4
Helmreich	X	0	0	0	0	X	X	0	X	0	X	0	0	0	0	0	5
Hitchings	X	X	X	X	X	X	X	X	X	0	X	X	X	X	0	X	14
Kadar	X	0	0	0	0	0	X	0	X	0	0	0	0	0	0	0	5
Katz	X	X	X	X	X	X	X	X	X	0	X	0	0	0	X	0	11
Lee JH	X	X	X	X	X	0	X	X	X	0	X	0	X	X	0	X	11
Maddox	X	0	X	X	X	0	0	0	X	0	X	0	X	X	0	0	7
McKay/Bowie	X	X	X	X	X	X	X	X	0	X	0	0	0	0	0	0	10
McVeigh	X	0	0	0	X	0	0	X	X	0	X	0	0	0	0	0	7
Meeks	X	0	0	0	X	X	X	X	X	0	X	0	0	0	0	0	8
Nolan	X	X	X	X	0	X	X	X	X	0	X	0	X	X	0	0	10
Pagano	X	X	X	X	X	0	X	X	X	0	X	0	X	X	X	0	11
Spath	X	0	X	X	0	X	0	X	0	0	0	0	0	0	0	0	6
Spigelman	X	0	0	0	0	0	0	0	0	0	0	X	X	X	0	0	3
Stekelenburg	X	0	X	X	X	0	0	X	X	0	X	X	X	X	0	0	9
Strayer	X	X	X	X	X	X	0	0	X	0	X	0	X	X	0	0	10
Thielen	X	X	0	0	X	0	0	0	0	0	X	0	X	X	0	X	7
Thompson	X	0	0	0	X	0	0	X	0	0	X	0	0	0	0	0	5
Calder/Forster	X	X	X	X	X	0	X	X	X	0	X	X	X	X	0	X	13
Cosby	X	0	0	0	X	0	X	0	0	0	0	X	0	0	0	0	5
Lovett	X	X	X	X	X	0	X	X	0	0	X	X	0	0	0	0	9
Reznek/Jepson	X	0	0	0	X	X	X	X	X	0	X	0	X	X	X	X	11
Carbo	X	X	X	X	X	X	0	0	X	0	X	X	X	X	0	X	12
Pierluissi	X	0	X	X	X	0	0	X	X	0	0	0	0	0	0	0	6
Wu/Provonost	X	X	X	X	X	X	X	X	X	X	X	X	0	0	0	X	14
Vincent	X	X	0	0	X	X	X	0	0	0	0	0	X	X	0	X	8
Wolochynowych	X	0	0	0	X	0	X	X	0	0	0	0	0	0	0	0	6

## Discussion

Our systematic review shows that, in the first two decades since the IOM report calling for improved safety systems, there have been few articles outlining a comprehensive clinical care review process. Additionally, most articles discuss their care review systems in the context of describing an aspect of their process, or corresponding improvement initiative.

Systems analysis—defined as the assessment of the effects of external forces such as policies, workflows, and software such as the electronic medical record on the critical event—was the most commonly identified care review process characteristic. Many identified articles describe the importance of evaluating how a person works within a system, rather than in isolation, to identify improvement opportunities. Assuming individuals are properly motivated with benign intent, looking at the system surrounding, the care avoids an antagonistic approach and supports the IOM’s underlying reasons for calling for care review processes—to prevent future errors.

Similarly, standardized processes and structured case classification were frequently discussed in the literature. To meet the IOM’s recommendations for creating care review “systems,” having a standardized process that uses structured classification is likely necessary. Without standardization, reviews would likely be sporadic, inefficient, and challenging to implement and subsequently inform future practice. Without structured classification, one could assume that conclusions would also be difficult to interpret.

Although some of these characteristics were common among reported care review systems, others are only rarely reported. Recognition of excellence and blinding of cases were reported in just five (14%) of the reports. Institutions that recognize excellence while performing care reviews were supportive of the practice, and one can understand why this would support the culture needed to have an effective care review system, and perhaps designers of future care review systems may wish to consider implementing this component. Similarly, anonymous review, or blinding, is intended to reduce bias and may allow a more objective review of each case. However, its infrequent mention may be indicative of unpublished prior experiences that may have supported avoiding this practice. From our experience, these are controversial topics, and future work is needed to understand the effects of specific characteristics on the overall care review process.

One additional characteristic that review processes must be supported by a functioning organizational system should receive particular attention. Although this was specifically identified by only 19 organizations, the downstream benefit to reviewing an episode of care and making recommendations for change in a non-functional system is likely lost. Key stakeholders in the process (physicians, nurse practitioners, physician assistants, nursing staff, support staff, etc.) are seemingly necessary for the care review process, and the administrative and leadership structure must be supportive of recommendations for change after care review is completed. This combination is strongly conducive to a process that engenders trust from the care team, which in turn bolsters the system as cases are referred for review, and staff engage in further problem solving.

### Limitations

The articles evaluated come from a variety of settings—from consulting firms to in situ care review systems. Some authors strove to describe a comprehensive local practice, while others focused primarily on a particular component of a larger system. This heterogeneity limits the generalizability as the variability from one system to another may indicate institution- or system-specific adaptations to facilitate the process. A solution for one setting may not represent a good solution for another. We included articles from institutions and consulting firms describing or self-reporting care review systems, and it is not possible to know the true effectiveness of the processes described when removed from clinical context. It may be that there is an over-emphasis on some areas of care review believed to be ideal that are not practiced as described, and also possible that not all aspects of a process are represented. Particularly in the articles that discuss the care review process as the context for a specific project, it is possible that not all the details on the over-arching system of care review in place are described resulting in abstraction of domains in what is an incomplete description.

The domains we used during abstraction were determined by screening the included articles and supplemented by expert opinion. It is possible that there are additional variables that are more important, but less common, and were not included in our analysis. It is possible that a care review process we reviewed may include some of the 16 characteristics but did not specifically mention them in the articles reviewed. Additionally, the qualitative nature of the abstraction and interpretation of each item definitions are complex and may lead to less reliable results.

In an effort to reduce the effects of bias and definition complexity, all articles were reviewed in duplicate—both for inclusion in the study as well as abstraction of data. Disagreements were resolved by discussion and consensus for article inclusion and adjudicated by a third reviewer for the domain abstraction.

## Conclusion

Despite increased discussion among institutions such as IOM and the National Patient Safety Foundation, in the last 16 years, there have been relatively few publications describing clinical care review processes and no clear evidence of a cultural shift to embrace clinical care review in an organized fashion. We have identified 16 domains of focus in a care review process and found that the approach to care review is highly variable as represented in the literature.

### Future research

The effects of different aspects of care review processes have not been well studied. This presents an opportunity to evaluate processes that are present in many hospitals and health systems and identify truly effective, rather than simply common, practices, as identified within.

## Additional file

Additional file 1: Search strategy. (DOCX 14 kb)
