# Supplementary material for: Clinical care review systems in healthcare: a systematic review
Source: Int J Emerg Med. 2018 Feb 8;11:6. doi: 10.1186/s12245-018-0166-y (PMC5805667; doi:10.1186/s12245-018-0166-y)
Supplement: Supplementary file 1 — Search strategy. (DOCX 14 kb) [file 12245_2018_166_MOESM1_ESM.docx]

**Additional file 1**: Search strategy

1.     peer review/ or peer review, health care/

2.     ((peer or case or care) adj (review* or assess*)).mp. [mp=title, abstract, heading word, drug trade name, original title, device manufacturer, drug manufacturer, device trade name, keyword]

3.     1 or 2

4.     medical staff, hospital/ or "internship and residency"/ or students, medical/ or exp specialties, medical/ or exp specialties, surgical/ or exp physicians/

5.     (physician* or doctor* or "medical student" or attending or surgeon* or "general practitioner" or "family physician" or hospitalist*).mp. [mp=title, abstract, heading word, drug trade name, original title, device manufacturer, drug manufacturer, device trade name, keyword]

6.     3 and (4 or 5)

7.     6 not (publish* or journal* or author* or "editorial board*").mp. [mp=title, abstract, heading word, drug trade name, original title, device manufacturer, drug manufacturer, device trade name, keyword]

8.     exp Medical Errors/ or competen*.mp. or clinical competence/ or (critical* adj2 apprais*).mp. [mp=title, abstract, heading word, drug trade name, original title, device manufacturer, drug manufacturer, device trade name, keyword]

9.     (mistake* or missed or misdiagnos* or mishandl* or overlooked or misinterpret* or appropriate* or inappropriate*).mp. [mp=title, abstract, heading word, drug trade name, original title, device manufacturer, drug manufacturer, device trade name, keyword]

10.   ("critical incident" or incident*1 or "self-report*" or discrepanc* or audit*).mp. or mo.fs. or mortality.mp. or morbidity.mp. or "near miss*".mp. [mp=title, abstract, heading word, drug trade name, original title, device manufacturer, drug manufacturer, device trade name, keyword]

11.   (feedback* or safety or harm* or adverse or hazard* or unsafe* or "root cause" or system*1 or complicat* or death*).mp. or quality assurance, health care/ or "quality improve*".mp. or risk.mp. [mp=title, abstract, heading word, drug trade name, original title, device manufacturer, drug manufacturer, device trade name, keyword]

12.   ((performance or outcome* or system* or practice*) adj3 (change or improv* or assess*)).mp. [mp=title, abstract, heading word, drug trade name, original title, device manufacturer, drug manufacturer, device trade name, keyword]

13.   (professional adj2 development).mp. [mp=title, abstract, heading word, drug trade name, original title, device manufacturer, drug manufacturer, device trade name, keyword]

14.   exp education, medical/ or curricul*.mp. or ed.fs. or educat*.mp. or professional*.mp. [mp=title, abstract, heading word, drug trade name, original title, device manufacturer, drug manufacturer, device trade name, keyword]

15.   or/8-14

16.   "quality of health care"/ or "outcome and process assessment (health care)"/ or "outcome assessment (health care)"/ or "process assessment (health care)"/ or health care evaluation mechanisms/

17.   15 or 16

18.   3 and 17

19.   7 and 17

20.   18 or 19

21.   20 not ((publish* or journal* or author* or "editorial board*" or publication*).mp. or periodicals as topic/ or submission*.mp.)

22.   *peer review/ or *peer review, health care/ or ((peer or case or care) adj (review* or assess*)).ti.

23.   21 and 22

24.   remove duplicates from 23
